# Genetic Transformation of the Marine Oleaginous Microalga, *Marinichlorella* sp. NKG400014

**DOI:** 10.1007/s10126-025-10473-6

**Published:** 2025-06-05

**Authors:** Ryota Kumakubo, Kento Sagawa, Tsuyoshi Tanaka

**Affiliations:** Division of Biotechnology and Life Science, Institute of Engineering, Tokyo University of Agriculture and Technology, 2-24-16 Naka-Cho, Koganei, Tokyo, 184-8588 Japan

**Keywords:** *Marinichlorella* sp. NKG400014, Biofuel production, Electroporation, Genetic transformation, Endogenous promoter

## Abstract

Microalgae attract significant attention as promising biofuel sources. However, lack of an efficient genetic transformation system hinders their use in advanced genetic engineering applications. We previously identified the marine oleaginous microalgal strain, NKG400014, from our marine microalgal culture collection via Nile red screening. Subsequent phylogenetic analysis revealed that strain NKG400014 belonged to the new genus, *Marinichlorella*. To date, successful genetic transformation of *Marinichlorella* species has not been reported. In this study, we aimed to investigate the growth characteristics of NKG400014 to clarify its oleaginous features as a biofuel producer. Then, we established a technique for NKG400014 genetic transformation via electroporation and optimized the key parameters. Two selectable markers (hygromycin phosphotransferase and neomycin phosphotransferase II genes) were used to assess the transformation efficiency under endogenous and exogenous promoters. Endogenous promoters of *Marinichlorella* sp. were more efficient for transformation than its exogenous promoters. To the best of our knowledge, this study represents the first successful genetic transformation of *Marinichlorella* sp.. Further development of genetic tools and metabolic engineering approaches can enhance the lipid productivity of this strain, facilitating the economic production of microalgal biofuels.

**Supplementary Information:**

The online version contains supplementary material available at 10.1007/s10126-025-10473-6.

## Introduction

Recently, microalgae have gained significant attention as promising sources for sustainable food, feed, biochemical, and biofuel production. Specifically, previous studies have investigated biofuel production using microalgae to reduce greenhouse gas emissions and identify potential renewable energy sources (Occhipinti et al. 2024; Abdullah et al. 2024). To date, several oleaginous microalgae have been identified and characterized for their biofuel production potential (Xue et al. 2021). Advances in metabolic engineering, including genome editing and genetic transformation technologies, have significantly improved the microalgal lipid productivity, enhancing the economic feasibility of microalgal biofuel production (Lu et al. 2021; Yang et al. 2016). Therefore, new tools and strategies are needed to obtain genetically engineered microalgae that can be used in molecular breeding via metabolic engineering.

Class Trebouxiophyceae is a major part of the green algal phylum Chlorophyta (Lemieux et al. 2014; Metz et al. 2019). *Chlorella* belongs to class Trebouxiophyceae. Particularly, *Chlorella vulgaris* and *C. sorokiniana* have been extensively studied for their biofuel production potential (Feng et al. 2011; Li et al. 2007; Xiong et al. 2008). However, most algae of the order Chlorellales studied to date have been isolated from freshwater environments, and the application of genetic transformation techniques to marine or salt-tolerant strains remains limited (Gu et al. 2023). Use of seawater for mass microalgal cultivation has several advantages (Maeda et al. 2018). As the global freshwater demand is projected to increase by 30% by 2050 (Beddington 2009), marine microalgae can be used to conserve freshwater resources and promote sustainable biofuel production systems (Boretti and Rosa 2019; Mahata et al. 2022). Furthermore, use of seawater for mass cultivation prevents contamination by other microorganisms (Mooij et al. 2015). Recently, genus *Marinichlorella*, a close relative of *Chlorella*, was classified as a marine strain showing salt tolerance (Aslam et al. 2007). This genus has garnered attention for its high biomass productivity and lipid accumulation capacity, as well as its ability to grow in seawater, which offers the advantage of conserving freshwater resources (Aslam et al. 2007; Kuehnle et al. 2015; Sánchez-Alvarez et al. 2017). Therefore, the establishment of a genetic transformation method for the genus *Marinichlorella* would provide a valuable platform for seawater-based microalgal biotechnology. However, to date, no study has reported successful genetic transformation in genus *Marinichlorella*.

Various methods, such as particle bombardment, polyethylene glycol-mediated transformation, *Agrobacterium*-mediated transformation, and electroporation, have been developed for genetic transformation of *Chlorella* species (Yang et al. 2016). However, most transformation methods have been established for freshwater *Chlorella* spp., with only one report on the transformation of marine *Chlorella* (Gu et al. 2023). Promoter selection plays a crucial role in the stable transformation of *Chlamydomonas reinhardtii*, *Nannochloropsis* sp., and *Phaeodactylum tricornutum,* with various molecular tools developed for this specific purpose. Endogenous promoters ensure higher expression efficiency and stability than exogenous promoters (Schroda et al. 2000; Vieler et al. 2012; Watanabe et al. 2018). However, most studies have used cauliflower mosaic virus (CaMV) or *Chlamydomonas reinhardtii* promoters (Yang et al. 2016), with only a few using endogenous promoters (Gu et al. 2023; Kim et al. 2018; Shin et al. 2020) for *Chlorella* species.

We previously identified marine oleaginous microalgae from a marine microalgal culture collection (Matsumoto et al. 2010). Among them, the marine green microalga strain NKG400014 was identified as a high oil-producing strain from a collection of 1,393 marine microalgal strains screened via Nile red staining (Matsumoto et al. 2010). Subsequent phylogenetic analysis revealed that strain NKG400014 belonged to the new genus, *Marinichlorella*, although it was initially classified as a *Chlorella* sp. Development of an efficient genetic transformation system is crucial to improve the oil productivity and commercial application of this genus.

In this study, we established a method for the genetic transformation of the marine oleaginous microalga, *Marinichlorella* sp. NKG400014, via electroporation. Various electroporation parameters, including voltage, pulse duration, and plasmid concentration, were optimized to determine the most effective conditions for gene delivery. Furthermore, antibiotic resistance gene expression was evaluated under both endogenous and exogenous promoters. Our results showed superior transformation efficiency under endogenous promoters.

## Materials and Methods

### Strains and Culture Conditions

Strain NKG400014 was cultured in the BG-11 medium prepared according to the formulation outlined by the Canadian Phycological Culture Centre (Centre 2025) and dissolved in artificial seawater (Tomita Pharmaceutical Co., Ltd., Tokushima, Japan) at 25 °C under 140 μmol photons/m^2^/s. Modified BG-11 was also prepared as nitrogen-depleted medium, in which NaNO₃ concentration was reduced from 1500 to 150 mg/L. Photon flux density at 400–700 nm was measured using the HD2302.01 luminometer with the LP471PAR probe (Delta OHM S.r.l, Caselle di Selvazzano, Italy). *Escherichia coli* EPI-300 was cultured in a lysogeny broth medium containing 50 μg/mL ampicillin (Sigma-Aldrich Co. LLC, St. Louis, MO, USA) or kanamycin (FUJIFILM Wako Pure Chemical Corporation, Osaka, Japan) at 37 °C.

### Phylogenetic Analysis

Microalgal cells (5 × 10^9^ cells) were harvested via centrifugation at 9,000 × *g* for 10 min at 4 °C, and genomic DNA was extracted from the biomass using the NucleoBond HMW DNA kit (Takara Bio, Shiga, Japan). Subsequently, *18S rRNA* gene of the strain NKG400014 was amplified from the genomic DNA via polymerase chain reaction (PCR) using the universal primers (Rowan and Knowlton 1995), 1500 F (5′-GGTGATCCTGCCAGTAGTCATATGCTTG-3′) and 1500R (5′-GATCCTTCCGCAGGTTCACCTACGGAAACC-3′), and Prime STAR Max DNA polymerase (Takara, Osaka, Japan). PCR was conducted as follows: Initial denaturation at 98 °C for 1 min, followed by 35 cycles of 98 °C for 10 s, 65 °C for 15 s, and 72 °C for 30 s, with a final extension at 72 °C for 7 min. For Sanger sequencing, 528 F primer (Gunderson et al. 1986) was used in addition to the 1500 F and 1500R primers. The 18S rDNA sequence of NKG400014 (1,687 bp) was further subjected to phylogenetic analysis. Other nucleotide sequences for phylogenetic analysis were obtained from the National Center for Biotechnology Information (http://www.ncbi.nlm.nih.gov; Table S1). Finally, a phylogenetic tree was constructed using the neighbor-joining method, excluding gaps caused by pairwise deletion, with the MEGA 11.0.13 software (Tamura et al. 2021). Bootstrap values were calculated for 2,000 replicates.

### Evaluation of Growth Characteristics

Growth experiments were conducted in the BG-11 or modified BG-11 medium. First, small volume cultures (80-mL cultures in test tubes) were used to evaluate the effects of light intensity (150, 500, and 1,000 µmol photons/m^2^/s) and temperature (25, 30, 35, and 40 °C) on biomass production. Based on the results, 1-L cultures in flat-shaped flasks were further used to estimate the maximum oil productivity with aeration at 0.8 L/L/min with 6% CO₂. After cultivation, the cells were harvested, lyophilized, and subjected to biomass and oil quantification by weighing, as previously described (Nomaguchi et al. 2018; Aketo et al. 2020b). At 24-h intervals, 1 mL of culture medium was collected to determine the cell density and nitrate and phosphate concentrations. Nitrate and phosphate concentrations in the microalgal cultures were measured via high-performance liquid chromatography. Cells and insoluble materials were removed from the microalgal culture media using a membrane filter (Millex Filter Unit 0.22 μm; [Merck Millipore, Darmstadt, Germany]). Subsequently, the supernatant was subjected to high-performance liquid chromatography with an anion-exchange column to measure the nitrate concentration, as previously described (Aketo et al. 2020a). Phosphate concentration was determined using the molybdenum blue method, as previously described (Aketo et al. 2021).

### Quantification of Carbohydrate, Protein, and Oil Contents

Next, phenol–sulfuric acid method was used to quantify the saccharide content (DuBois et al. 1956). Lyophilized cells were suspended in 500 μL of ultrapure water at a concentration of 0.5 mg/mL, followed by the addition of 500 μL of 5% (w/v) phenol solution and 2.5 mL of concentrated sulfuric acid. The samples were incubated at 25 °C for 30 min, and absorbance at 490 nm was measured using a microplate reader (SH-9000; Corona Electric, Ibaraki, Japan). Protein content was determined using the BCA Protein Assay Kit (Thermo Fisher Scientific, Waltham, MA, USA). Absorbance at 562 nm was measured using a microplate reader. Then, total lipids were extracted using chloroform/methanol (2:1, v/v), as previously described (Nomaguchi et al. 2018).

### Plasmid Construction

Plasmid pCAMBIA1302 (ab275760; Abcam, Cambridge, UK), which expresses hygromycin phosphotransferase (*hpt*) under the control of the CaMV 35S promoter, was used to optimize the electroporation conditions. To compare various promoter activities, pICSL11055 (#68,252; Addgene, Watertown, MA, USA), which expresses neomycin phosphotransferase II (*nptII*) under the control of the CaMV 35S promoter, was used as the backbone vector. This vector was used to construct plasmids incorporating the 500-bp upstream regions of the following genes (Table 1): Ribulose-1,5-bisphosphate carboxylase small subunit II (*RBCS2*), photosystem I reaction center subunit II (*psaD*), and glyceraldehyde-3-phosphate dehydrogenase (*GAPDH*). *GAPDH* is a well-known housekeeping gene, and its upstream region is an effective promoter in *Dunaliella salina* and *Fistulifera solaris* (Jia et al. 2012; Nojima et al. 2013). In *Chlamydomonas reinhardtii*, *RBCS2* promoter exhibits strong activity (Lumbreras et al. 1998), whereas *psaD* promoter functions as a light-responsive promoter (Crozet et al. 2018). GAPDH, RBCS2, and psaD proteins in NKG400014 were searched using the Basic Local Alignment Search Tool for proteins with the query sequences of *Chlamydomonas reinhardtii* RBCS2 (UniProtKB/Swiss-Prot: P08475.1), GAPDH (UniProtKB/Swiss-Prot: P49644.1), and psaD (UniProtKB/Swiss-Prot: Q39615.1) against the predicted ones from the draft genome sequence of NKG400014 (unpublished data). E-value threshold < 10⁻^4^ was applied. To amplify the 500-bp upstream regions from the start codons of the selected genes, primers were designed (Table S2), and PCR was performed using the genomic DNA extracted from NKG400014 during the stationary phase using the NucleoBond HMW DNA Kit (Macherey–Nagel, Düren, Germany).

**Table 1:** Construction of plasmids with various promoters

Plasmid	Promoters	Source of promoters	Antibiotic-resistant genes
pCAMBIA1302	*CaMV*	Cauliflower mosaic virus	*hpt*
pICSL-NPT/CaMV	*CaMV*	Cauliflower mosaic virus	*nptII*
pICSL-NPT/g6588	*RBCS2_1*	*Marinichlorella* sp. NKG400014	*nptII*
pICSL-NPT/g6589	*RBCS2_2*	*Marinichlorella* sp. NKG400014	*nptII*
pICSL-NPT/g2132	*GAPDH_1*	*Marinichlorella* sp. NKG400014	*nptII*
pICSL-NPT/g4405	*GAPDH_2*	*Marinichlorella* sp. NKG400014	*nptII*
pICSL-NPT/g6321	*psaD_2*	*Marinichlorella* sp. NKG400014	*nptII*

PCR was performed as follows: Initial denaturation at 98 °C for 1 min, followed by 35 cycles of 98 °C for 10 s, 55 °C for 15 s, and 72 °C for 30 s, with a final extension at 72 °C for 7 min. The CaMV 35S promoter sequence was amplified from pICSL11055 under the same PCR conditions. Additionally, a 5441-bp fragment containing the *nptII* gene sequence was amplified from pICSL11055 using the pICSL11055_F and R primers as a backbone. The resulting PCR products were purified using the QIAquick Gel Extraction Kit (Qiagen, Hilden, Germany) or QIAquick PCR Purification Kit (Qiagen). The purified fragments were further assembled using the NEBuilder HiFi DNA Assembly Master Mix (New England Biolabs, Tokyo, Japan) at 50 °C for 1 h. The assembled plasmids were introduced into electrocompetent *E. coli* EPI-300 cells via electroporation. Finally, plasmid sequences were verified via Sanger sequencing to confirm efficient plasmid construction.

### NKG400014 Transformation via Electroporation

NKG400014 was cultured in the BG-11 medium, and 5 × 10^5^ cells in the logarithmic growth phase were harvested via centrifugation at 800 × *g* for 10 min at 25 °C. Cell pellet was washed thrice with distilled water. Washed cells were resuspended in 45 µL of a solution containing 0.2 M mannitol and 0.2 M sorbitol and incubated on ice for 40 min. Subsequently, an equal volume (45 µL) of a second solution containing 0.2 M mannitol, 0.2 M sorbitol, 0.08 M KCl, 0.005 M CaCl₂, and 0.01 M HEPES (pH 7.2) was added. Then, UltraPure Salmon Sperm DNA Solution (Thermo Fisher Scientific) and plasmid DNA were added to final concentrations of 150 and 30 µg/mL, respectively. The cell–plasmid suspension was transferred to a 0.2-cm cuvette (NEPA GENE, Chiba, Japan) and incubated on ice for 10 min. Electroporation was performed using Gene Pulser Xcell (Bio-Rad, Hercules, CA, USA). Immediately after pulsing, the cells were resuspended in 1 mL of BG-11 medium and incubated in the dark at 25 °C for 24 h for recovery. Following incubation, the cells were spread onto BG-11 agar plates with 500 µg/mL of G-418 or 600 µg/mL of hygromycin and cultured at 100 µmol photons/m^2^/s for 21 d at 25 °C to facilitate colony formation.

### PCR Analysis of the Selection Marker Gene

Hygromycin-resistant colonies were suspended in 20 µL of distilled water and heated at 99 °C for 15 min to extract the DNA from the cells. A 50-μL PCR mixture contained 2 × PrimeSTAR Max Premix (Takara Bio), 0.5 μM primers (HygR_fwd; 5′-TGGGGCGTCGGTTTCCACTA-3′, HygR_rev; 5′-TGCGCGATTGCTGATCCCCA-3′), and 10 µL of extracted supernatant. Amplification was performed using a thermal cycler (Applied Biosystems, Foster City, CA, USA) under the following conditions: Initial denaturation at 98 °C for 1 min, followed by 35 cycles of 98 °C for 10 s, 55 °C for 5 s, and 72 °C for 5 s, with a final extension at 72 °C for 7 min. PCR products were analyzed via 1% agarose gel electrophoresis using the Tris–acetate-EDTA buffer at 100 V for 30 min. Subsequently, DNA fragments were visualized using a UV transilluminator after staining with Gel Red (Biotium, Fremont, CA, USA).

### Statistical Analyses

Data are represented as the mean ± standard deviation of the mean of triplicate samples. Significant differences between means due to temperature and light intensity were determined via two-way analysis of variance, followed by Duncan’s multiple-range test. All statistical analyses were conducted using the R software (version 4.4.2) with the stats (version 4.4.2; base R) and agricolae (version 1.3.7) packages (de Mendiburu 2023). Statistical significance was set at p < 0.05.

## Results and Discussion

### Phylogenetic Analysis of the Strain NKG400014

**Fig. 1:**
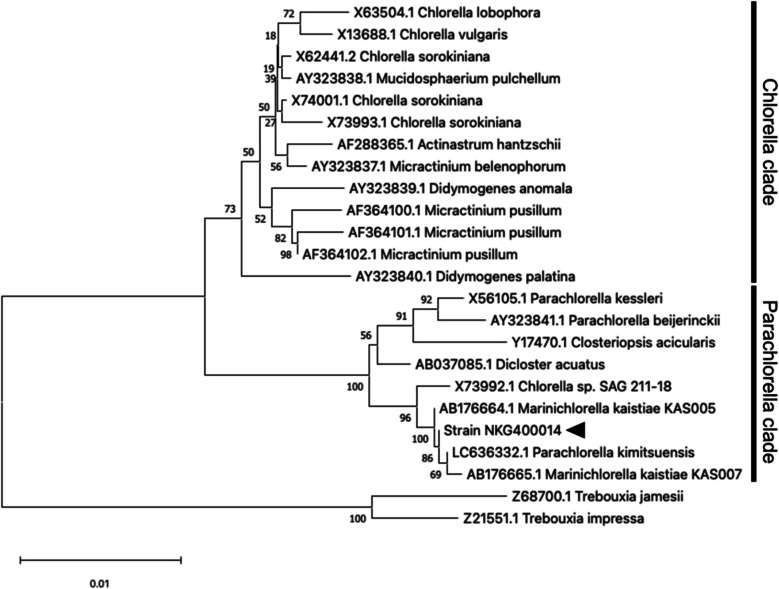
Phylogenetic tree constructed based on the 18S rDNA sequences of NKG400014 and its closest group representatives. Scale bar indicates the phylogenetic distance (0.01 substitutions per sequence position). Bootstrap values shown on the nodes were recovered via neighbor-joining analysis

Phylogenetic analysis revealed that NKG400014 clustered within the *Parachlorella* clade of order *Chlorellales* (Fig. 1). Within this clade, five strains, including NKG400014, comprised a monophyletic group with a high bootstrap value of 100%. Among them, *M. kaistiae* KAS005 and KAS007 were recently separated from the *Parachlorella* clade, which is isolated from marine environments and composed of salt-tolerant species (up to 7.5% NaCl [w/v]) (Aslam et al. 2007). *P. kimitsuensis* is isolated from marine and brackish environments (Ota et al. 2023), whereas other *Parachlorella* spp. (*P. kessleri*, *P. beyerinckii*, and *P. hussii*) are isolated from freshwater (Krienitz et al. 2004; Bock et al. 2011). *Chlorella* sp. SAG 211–18 strain was reportedly isolated from freshwater (cliff pool); however, owing to the proximity of the cliff pool to the coast (Huss et al. 1999), this strain could have been isolated from a marine environment instead. Here, NKG400014, maintained in the marine microalgal culture collection at our university, was isolated from a coastal area. As all strains in this group are isolated from marine environments (Aslam et al. 2007), the five strains in the monophyletic group should be classified as *Marinichlorella* species. NKG400014 showed the highest 18S rDNA sequence relatedness with *P. kimitsuensis* and *M. kaistiae* KAS005 (99.94% similarity). The branch point of NKG400014 and its closest relatives was supported by 100% recovery in the bootstrap analysis. These results suggest the strain NKG400014 as a new species in the *Marinichlorella* genus.

### Oil Accumulation by Strain NKG400014

**Fig. 2:**
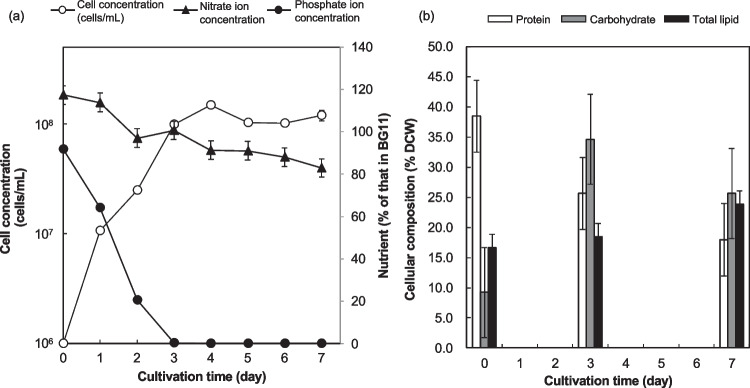
Growth and compositional analysis of the NKG400014 strain. (**a**) Growth curve of the cell and nitrate and phosphate concentrations. (**b**) Cellular composition of total lipids, carbohydrates, proteins. NKG400014 was cultured at 1,000 µmol photons/m^2^/s for seven days at 35 °C in the BG-11 medium. Error bars represent the standard error (SE) of the mean (n = 3)

Growth characteristics of NKG400014 were investigated to quantitatively clarify its oil productivity. First, basic culture conditions were investigated under different temperature and light conditions using 80-mL cultures in a test tube to maximize biomass production (Table 2). Biomass production first increased from 25 to 35 °C and then decreased at 40 °C. Maximum biomass production (3.94 ± 0.03 g/L) was observed at 1000 µmol photons/m^2^/s. Oil content was below 20% under these conditions. Subsequently, *Marinichlorella* sp. NKG400014 was cultured in 1-L flat-shaped flasks at 35 °C and 1000 µmol photons/m^2^/s to evaluate its oil productivity (Fig. 2). Cell concentration reached a plateau after three days (Fig. 2a). Growth arrest occured upon phosphorus depletion in the culture medium. Biomass production and oil content were 3.89 ± 0.33 g/L and 23.9 ± 1.2%, respectively, at seven days (Fig. 2b). Oil content was slightly higher in the flat-shaped flasks than in the test-tube flasks (Table 2), possibly due to the enhanced light penetration promoting carbon flux redirection from carbohydrates to lipid biosynthesis in the flat-shaped flasks. Indeed, a temporal increase in lipid content was accompanied by a decrease in both carbohydrate and protein contents. Maximum oil productivity was 132.7 ± 12.0 mg/L/day. Oil productivity of *Marinichlorella* sp. NKG400014 were comparable to those of other oleaginous microalgae, such as *Chlorella* sp., *F. solaris*, and *N. oceanica* (Table 3). Furthermore, oil content of NKG400014 reached up to 37.0 ± 1.9% under nitrogen-depleted conditions (Fig. S1). These results highlight the oleaginous properties of *Marinichlorella* sp. NKG400014.

**Table 2:** Effects of temperature and light intensity on biomass production by and oil content of the NKG400014 strain

Temperature (°C)	Light intensity (µmol photons/m^2^/s)	Biomass production (g/L)	Oil content (%w/w)
25	150	2.37 ± 0.01 ^Cc^	19.0 ± 0.6
30	150	2.68 ± 0.05 ^Ac^	17.3 ± 0.3
35	150	2.84 ± 0.02 ^Bc^	19.1 ± 1.2
40	150	0.56 ± 0.08 ^Dc^	ND
30	500	3.49 ± 0.15 ^Ab^	18.4 ± 1.4
30	1,000	3.94 ± 0.07 ^Aa^	18.6 ± 0.7

Unless otherwise stated, the growth conditions were as follows: BG-11 medium and an air flow rate of 0.8 L/L/min (2% CO_2_).*ND* Not Determined. The measurement was not conducted due to insufficient biomass for oil extraction.Data are given as means ± S.D., n = 3. Different uppercase letters indicate significant differences among temperature conditions, while different lowercase letters indicate significant differences among light intensity conditions (two-way ANOVA, Duncan’s test; p < 0.05). No significant difference was observed in oil content among culture conditions

**Table 3:** Comparison of the biomass and oil productivities and oil contents of different marine microalgae

Microalgae species	Biomass productivity (mg/L/day)	Oil content (%)	Oil productivity (mg/L/day)	References
*Cheatocerous muelleri*	110	39	42.9	(Andrew et al. 2022)
*Neochloropsis* sp.	210.0	29.6	61.0	(Rodolfi et al. 2009)
*Isochrysis galbana*	290.0	30.1	87.3	(Andrew et al. 2022)
*Chlorella* sp.	339.0	32.4	110.0	(Hsieh and Wu 2009)
*Marinichlorella* sp. NKG400014	555.1 ± 82.9	37.0 ± 1.9	132.7 ± 20.8	This study
*Fistulifera solaris* JPCC DA0580	485.7	55.0	142.7	(Liang et al. 2014)
*Nannochloropsis oceanica* IMET1	300.6 ± 20.3	52.92 ± 1.04	158.76 ± 13.83	(Ma et al. 2014)

### *Marinichlorella *sp. NKG400014 Transformation via Electroporation

**Fig. 3:**
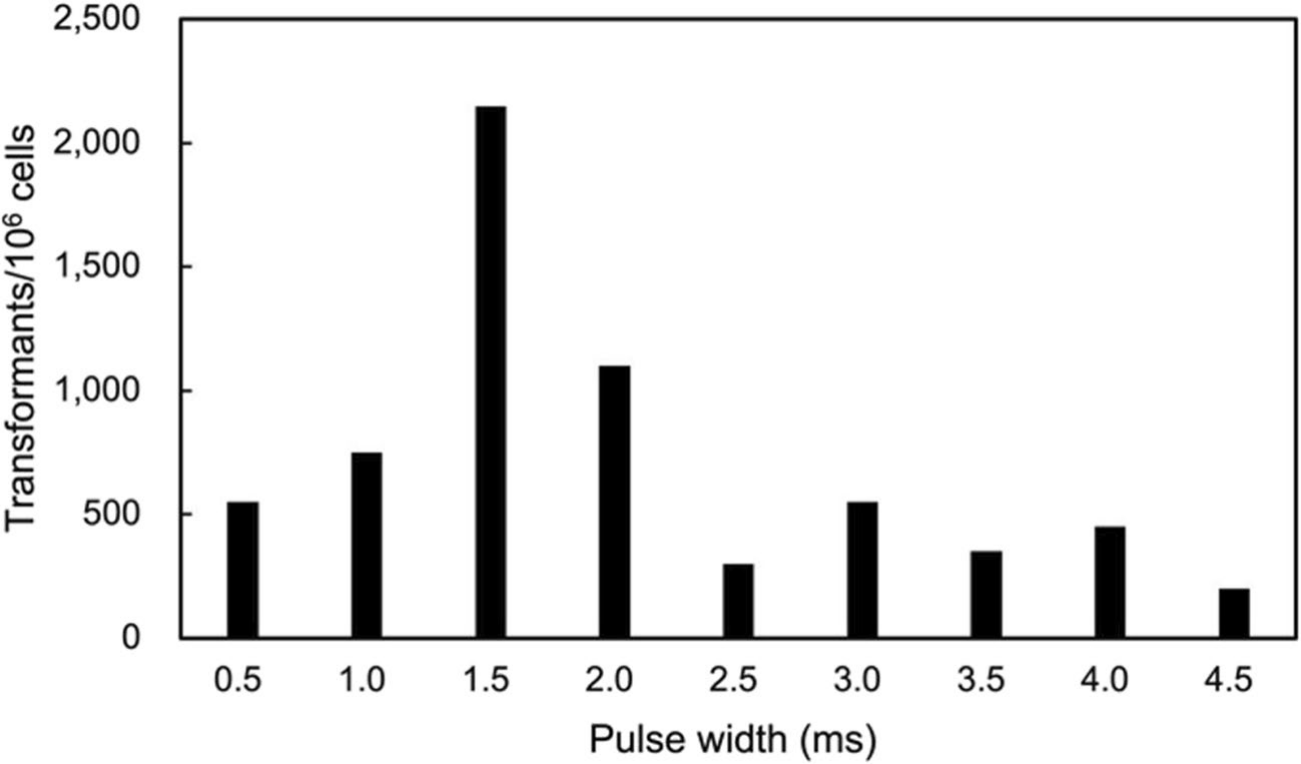
Numbers of transformant clones obtained under varying pulse width conditions via electroporation. Plasmid pCAMBIA1302 was introduced into NKG400014 cells, and transformant clones were selected using the hygromycin-containing agar medium. Presence of hygromycin phosphotransferase (*hpt*) was verified via polymerase chain reaction (PCR). Electroporation parameters: Field strength = 3.3 kV/cm and plasmid concentration = 30 µg/mL

**Fig. 4:**
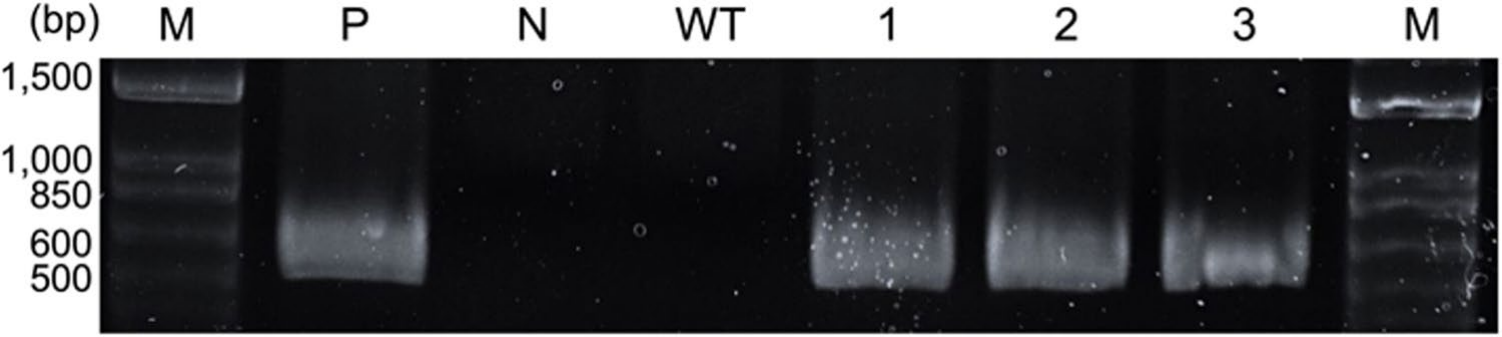
PCR amplification of *hpt* (547 bp) in the transformants. Lane M: 1-kb maker; lane P: pCAMBIA1304 (positive control); lane N: Pure water (negative control); lane WT: Wild-type; lanes 1–3: Transformants

NKG400014 transformation was performed via electroporation using the pCAMBIA1302 plasmid, which expresses the hpt gene under the control of the CaMV 35S promoter. No clear relationship was observed between the number of transformants and electric field strength (1.8–5.3 kV/cm) at 3.5 ms of pulse width. Effect of pulse width on transformation was investigated under a moderate potential (3.3 kV/cm field strength). Antibiotic-resistant colonies were obtained under several pulse conditions, and all colonies were subjected to PCR analysis. Clones that tested positive for the *hpt* gene were defined as confirmed transformants. Histograms plotting the number of transformants showed a wide distribution with a highest peak at 1.5 ms (Fig. 3, Table S3). Short pulses were insufficient to induce effective gene transfer, whereas long pulses (> 2.5 ms) induced excessive cellular stress. The maximum transformation efficiency was 71.7 transformants/10⁶ cells with 1 μg of plasmid. Table 4 presents the transformation efficiencies of green microalgae upon electroporation. The transformation efficiency observed in this study is comparable to previously reported values in green algae. This supports the successful establishment of a transformation method for *Marinichlorella* sp. NKG400014. To evaluate the transgene stability, three *hpt*-positive clones were randomly selected, transformant cultures were passaged for six months, and integration of selection marker genes was confirmed via PCR. PCR detected the *hpt* gene in the clones cultured for six months (Fig. 4), demonstrating the stability of the introduced DNA in the transformants for at least six months.

**Table 4:** Transformation efficiency achieved in this study compared to those reported in previous studies reported studies

Microalgae species	Transformation efficiency (× 10^–6^ cells)	References
*Chlamydomonas reinhardtii*	720 ± 16	(Shimogawara et al. 1998)
*Chlorella vulgaris*	500 ± 18	(Chow and Tung 1999)
*Dunaliella salina*	190 ± 92	(Geng et al. 2003)
*C. ellipsoidea*	7500	(Bai et al. 2013)
*C. pyrenoidosa*	610.2 ± 43.8	(Run et al. 2016)
*Marinichlorella* sp. NKG400014	2150	This study

### Effects of Endogenous Promoters on NKG400014 Transformation

Generally, promoter selection is crucial for efficient microalgal transformation (Schroda et al. 2000; Vieler et al. 2012; Watanabe et al. 2018). Therefore, impact of an endogenous promoter on transformation efficiency was investigated in this study. Table 5 shows the numbers of antibiotic-resistant colonies of NKG400014 formed under endogenous and exogenous promoters. CaMV 35S promoter, commonly used for gene expression in microalgae, was used as an exogenous promoter. Although all promoters resulted in antibiotic-resistant colony formation, more antibiotic-resistant colonies were formed under endogenous promoters than under exogenous promoters. Although no remarkable changes were observed in the colony number, GAPDH_1 promoter resulted in the highest number of colonies. GAPDH promoter also demonstrates high transformation efficiency in *F. solaris* and *D. salina* (Jia et al. 2012; Nojima et al. 2013), serving as a valuable promoter. Overall, endogenous promoters effectively promoted stable gene expression in *Marinichlorella* sp..

**Table 5:** Numbers of G418-resistant colonies obtained after the introduction of vectors with different promoters

Promoters	Colony numbers per 10^5^ cells^a^	Number of clones grown in liquid medium/Number of randomly selected colonies
*CaMV35S*	37.3 (71,7,34)	14/20
*RBCS2_1*	95.7 (147,103,37)	28/44
*RBCS2_2*	127.7 (180,89,114)	8/9
*GAPDH_1*	165.0 (81,300,114)	30/48
*GAPDH_2*	129.7 (85,65,239)	6/8
*psaD*	113.7 (84,247,10)	21/27

a Colonies per 10^5^ cells represent the average number of G418-resistant colonies obtained from three independent replicates. Numbers in parentheses indicate colony counts from individual transformation experiments

## Conclusions

In this study, we developed a stable technique for *Marinichlorella* sp. NKG400014 transformation via electroporation. Use of endogenous promoters improved the transformation efficiency. Notably, marine strain NKG400014, possibly belonging to *Marinichlorella* sp., showed oleaginous features, with an oil productivity comparable to those of other oleaginous microalgae. To the best of our knowledge, this study is the first to demonstrate the successful genetic transformation of *Marinichlorella* sp.. Future studies should improve the genetic tools and metabolic engineering approaches to enhance the lipid productivity of this strain and facilitate its application for economic biofuel production.

## Supplementary Information

Below is the link to the electronic supplementary material. 

ESM1 (PDF 630 KB)

## Data Availability

The 18S rDNA sequence of strain NKG400014 is available in [GenBank/NCBI] under accession number PV419726.
