# Supplementary material for: Genetic Transformation of the Marine Oleaginous Microalga, Marinichlorella sp. NKG400014
Source: Mar Biotechnol (NY). 2025 Jun 5;27(3):91. doi: 10.1007/s10126-025-10473-6 (PMC12141415; doi:10.1007/s10126-025-10473-6)
Supplement: Supplementary file 1 — (PDF 630 KB) [file 10126_2025_10473_MOESM1_ESM.pdf]

Supplementary Information for

Genetic Transformation of the Marine Oleaginous Microalga, *Marinichlorella* sp. NKG400014

Ryota Kumakubo, Kento Sagawa, and Tsuyoshi Tanaka*

Division of Biotechnology and Life Science, Institute of Engineering, Tokyo University of Agriculture and Technology, 2-24-16 Naka-cho, Koganei, Tokyo, 184-8588, Japan

***Corresponding author:**

Tsuyoshi Tanaka

Tel: +81-42-388-7401

Fax: +81-42-385-7713

E-mail: tsuyo@cc.tuat.ac.jp

Table S1 GenBank/EMBL/DDBJ accession numbers

Accession no.	Species	Strain	References
AF288365	*Actinastrum hantzschii*	SAG 2015	(Krienitz et al. 2004)
X63504	*Chlorella lobophora*	Andreyeva 750-1	(Huss et al. 1999)
X73993	*Chlorella sorokiniana*	SAG 211-40	(Huss et al. 1999)
X62441	*Chlorella sorokiniana*	SAG 211-8k	(Huss et al. 1999)
X74001	*Chlorella sorokiniana*	Baslerová Prag A14	(Huss et al. 1999)
X73992	*Chlorella* sp.	SAG 211-18	(Huss et al. 1999)
X13688	*Chlorella vulgaris*	SAG 211-11	(Huss and Sogin 1990)
Y17470	*Closteriopsis acicularis*	SAG 11.86	(Krienitz et al. 2004)
AY323837	*Diacanthos belenophorus*	SAG 42.98	(Krienitz et al. 2004)
AB037085	*Dicloster acuatus*	SAG 41.98	(Krienitz et al. 2004)
AY323838	*Dictyosphaerium pulchellum*	SAG 222-2	(Krienitz et al. 2004)
AY323839	*Didymogenes anomala*	SAG 18.91	(Krienitz et al. 2004)
AY323840	*Didymogenes palatina*	SAG 30.92	(Krienitz et al. 2004)
AB176664	*Marinichlorella kaistiae*	KAS005	(Aslam et al. 2007)
AB176665	*Marinichlorella kaistiae*	KAS007	(Aslam et al. 2007)
AF364101	*Micractinium pusillum*	SAG 13.81	(Krienitz et al. 2004)
AF364102	*Micractinium pusillum*	SAG 48.93	(Krienitz et al. 2004)
AF364100	*Micractinium* sp.	SAG 72.80	(Krienitz et al. 2004)
X56105	*Parachlorella kesseleri*	SAG 211-11g	(Huss and Sogin 1990)
LC636332	*Parachlorella kimitsuensis*	NS001C	(Ota et al. 2023)
AY323841	*Parachlorella beyerinckii*	SAG 2046	(Krienitz et al. 2004)
AB176664	*Marinichlorella kaistiae*	KAS005	(Aslam et al. 2007)
AB176665	*Marinichlorella kaistiae*	KAS007	(Aslam et al. 2007)
Z21551	*Trebouxia impressa*	UTEX 892	(Sargent et al. 1988)
Z68700	*Trebouxia jamesii*	UBT-86.132E2	(Krienitz et al. 2004)

Table S2 List of primers

Primer name	Sequence (5'→3')	Propose
1500F	GGTGATCCTGCCAGTAGTCATATGCTTG	PCR amplification of 18S rDNA (Rowan and Knowlton 1995)
1500R	GATCCTTCCGCAGGTTCACCTACGGAAACC	PCR amplification of 18S rDNA (Rowan and Knowlton 1995)
528F	CGGTAATTCCAGCTCC	Sanger sequencing of 18S rDNA (Gunderson et al. 1986)
HygR_fwd	TGGGGCGTCGGTTTCCACTA	PCR amplification of *hpt* gene
HygR_rev	TGCGCGATTGCTGATCCCCA	PCR amplification of *hpt* gene
pICSL11055_fwd	atgattgaacaagatggattgc	Gibson assembly of pICSL-NPT/g2142, g4405, g6321, g6588, g6589 and CaMV35S
pICSL11055 _rev	gacctccggatccgaattc	Gibson assembly of pICSL-NPT/g2142, g4405, g6321, g6588, g6589 and CaMV35S
g2132up500_fwd	cgaattcggatccggaggtcAGGGTTTGGACGCATCG	Gibson assembly of pICSL-NPT/g2132
g2132up500_rev	aatccatcttgttcaatcatGTAGTCTGCACAGCATGGG	Gibson assembly of pICSL-NPT/g2132
g4405up500_fwd	cgaattcggatccggaggtcAGGTATGCCGCCTACATG	Gibson assembly of pICSL-NPT/g4405
g4405up500_rev	aatccatcttgttcaatcatAAACTGAGCAGCTGATGC	Gibson assembly of pICSL-NPT/g4405
g6321up500_fwd	cgaattcggatccggaggtcAACGCAGGAAACTTAGCAAC	Gibson assembly of pICSL-NPT/g6321
g6321up500_rev	aatccatcttgttcaatcatCTCGAAAATCTGCTCTTTCTTG	Gibson assembly of pICSL-NPT/g6321
g6588up500_fwd	cgaattcggatccggaggtcGGCGACAGCTTCGAACGG	Gibson assembly of pICSL-NPT/g6588
g6588up500_rev	aatccatcttgttcaatcatTGCGTGCAAAAGTTTTTTTGAGAGG	Gibson assembly of pICSL-NPT/g6588
g6589up500_fwd	cgaattcggatccggaggtcGTCTGCACTTGAGCGTAG	Gibson assembly of pICSL-NPT/g6589
g6589up500_rev	aatccatcttgttcaatcatTGCGTCAACAATGTGCAG	Gibson assembly of pICSL-NPT/g6589
CaMV35S_P_fwd	cgaattcggatccggaggtcTGAGACTTTTCAACAAAGG	Gibson assembly of pICSL-NPT/CaMV35S
CaMV35S_P_rev	aatccatcttgttcaatcatTGTCCTCTCCAAATGAAATG	Gibson assembly of pICSL-NPT/CaMV35S

Table S3 Numbers of CFU and transformant clones obtained under varying pulse width conditions via electroporation

Pulse width (ms)	Colony Formation Unit (CFU) per 10^4 cells[a]	Number of *hpt*-positive clones per 10^4 cells[b]
0	55, 30	10, 4
0.5	20, 37	9, 2
1.0	32, 42	13, 2
1.5	83, 19	41, 2
2.0	37, 38	17, 5
2.5	7, 30	6, 0
3.0	11, 12	5, 6
3.5	17, 24	5, 2
4.0	7, 40	3, 6
4.5	2, 39	2, 2

a: Colonies per 10^4 cells represent the average number of hygromycin-resistant colonies obtained from two independent replicates.

b: Presence of hygromycin phosphotransferase (*hpt*) was verified via polymerase chain reaction (PCR).

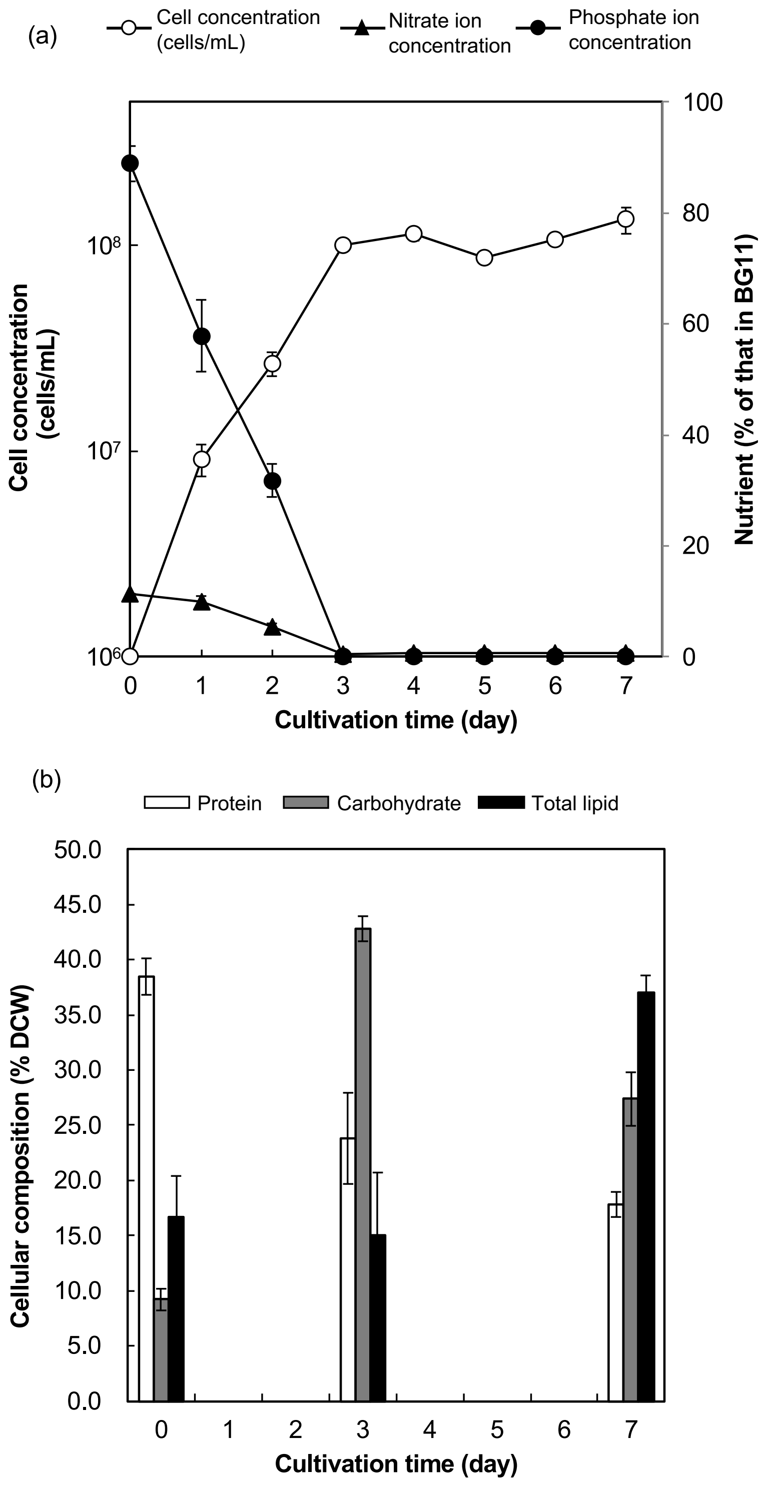

Fig. S1 Growth and compositional analysis of strain NKG400014. (a) Growth curves of cell concentration and nitrate ion concentrations, phosphate ion concentration. (b) Cellular composition of total lipids, carbohydrates, proteins. Strain NKG400014 was cultured at 35ºC, 1,000 µmol photons/m^2/s for 7 days in modified BG-11 medium. Error bars represent the standard error (SE) of the mean (n = 3).

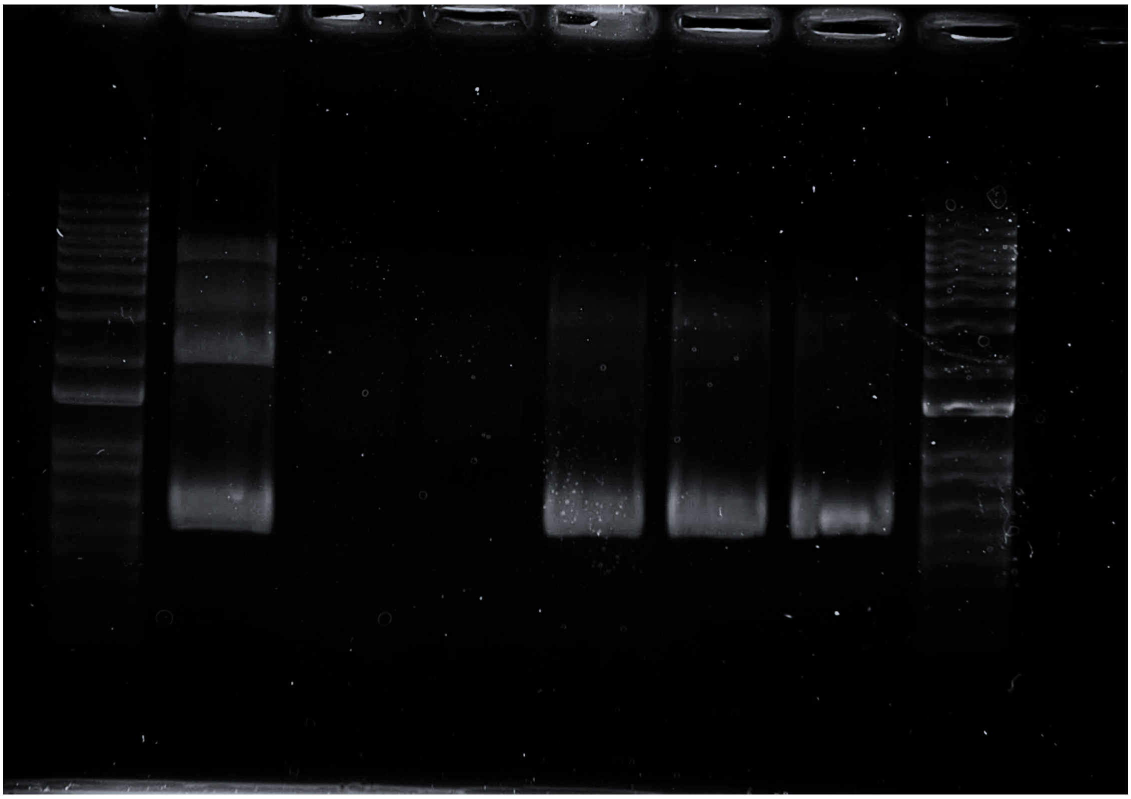

Fig. S2 Original image for Fig. 4.

References

Aslam Z, Shin W, Kim MK, Im WT, Lee ST (2007) *Marinichlorella Kaistiae* Gen. Et Sp. Nov. (Trebouxiophyceae, Chlorophyta) Based on Polyphasic Taxonomy1. J Phycol 43**:** 576-584

Gunderson J, Mccutchan T, Sogin M (1986) Sequence of the Small Subunit Ribosomal RNA Gene Expressed in the Bloodstream Stages of *Plasmodium berghei*: Evolutionary Implications 1. J Protozool 33**:** 525-529

Huss V, Sogin M (1990) Phylogenetic position of some *Chlorella* species within the Chlorococcales based upon complete small-subunit ribosomal RNA sequences. Journal of Molecular Evolution 31**:** 432-442

Huss VA, Frank C, Hartmann EC, Hirmer M, Kloboucek A, Seidel BM, Wenzeler P, Kessler E (1999) Biochemical taxonomy and molecular phylogeny of the genus *Chlorella* sensu lato (Chlorophyta). J Phycol 35**:** 587-598

Krienitz L, Hegewald EH, Hepperle D, Huss VA, Rohr T, Wolf M (2004) Phylogenetic relationship of *Chlorella* and *Parachlorella* gen. nov.(Chlorophyta, Trebouxiophyceae). Phycologia 43**:** 529-542

Ota S, Yoshimura K, Kosugi C, Kawano S (2023) Taxonomic and physiological studies of *Parachlorella kimitsuensis* sp. nov. (Trebouxiophyceae), which shows high ammonium tolerance. Algal Res 71

Rowan R, Knowlton N (1995) Intraspecific diversity and ecological zonation in coral-algal symbiosis. Proc Natl Acad Sci 92**:** 2850-2853

Sargent M, Zahn R, Walters B, Gupta R, Kaine B (1988) Nucleotide sequence of the 18S rDNA from the microalga *Nanochlorum eucaryotum*. Nucleic Acids Res 16**:** 4156
